# Thickness Dependence of Electronic Structure and Optical Properties of F8BT Thin Films

**DOI:** 10.3390/polym14030641

**Published:** 2022-02-08

**Authors:** Bita Ghasemi, Jakub Ševčík, Vojtěch Nádaždy, Karol Végsö, Peter Šiffalovič, Pavel Urbánek, Ivo Kuřitka

**Affiliations:** 1 Centre of Polymer Systems, Tomas Bata University in Zlin, Tr. Tomase Bati 5678, CZ-760 01 Zlin, Czech Republic; ghasemi@utb.cz (B.G.); j4sevcik@utb.cz (J.Š.); kuritka@utb.cz (I.K.); 2 Institute of Physics, Slovak Academy of Sciences, Dubravska cesta 9, SK-845 11 Bratislava, Slovakia; Vojtech.Nadazdy@savba.sk (V.N.); vegsok@gmail.com (K.V.); peter.siffalovic@savba.sk (P.Š.)

**Keywords:** F8BT, thin films, chain ordering, J- and H-aggregates, ER-EIS

## Abstract

Electronic devices based on polymer thin films have experienced a tremendous increase in their efficiency in the last two decades. One of the critical factors that affects the efficiency of polymer solar cells or light emitting devices is the presence of structural defects that controls non-radiative recombination. The purpose of this report is to demonstrate a non-trivial thickness dependence of optoelectronic properties and structure (dis)order in thin conductive poly(9,9-dioctyfluorene-alt-benzothiadiazole), F8BT, polymer films. The UV-Vis absorption spectra exhibited blue shift and peak broadening; significant changes in 0–0 and 0–1 radiative transition intensity was found in photoluminescence emission spectra. The density of state (DOS) was directly mapped by energy resolved-electrochemical impedance spectroscopy (ER-EIS). Satellite states 0.5 eV below the lowest unoccupied molecular orbital (LUMO) band were revealed for the thinner polymer films. Moreover, the decreasing of the deep states density in the band gap manifested an increment in the material structural ordering with increasing thickness. Changes in the ratio between crystalline phases with face-on and edge-on orientation of F8BT chains were identified in the films by grazing-incidence wide angle X-ray scattering technique. A thickness threshold in all investigated aspects of the films at a thickness of about 100 nm was observed that can be attributed to the development of J-H aggregation in the film structure and mutual interplay between these two modes. Although a specific structure–property relationship thickness threshold value may be expected for thin films prepared from various polymers, solvents and under different process conditions, the value of about 100 nm can be generally considered as the characteristic length scale of this phenomenon.

## 1. Introduction

The fact that “plastics” could be used to conduct electricity, discovered by Nobel Laureates A. Heeger, A. Mc Diarmid, and H. Shirakawa during their work on “conjugated organic polymer”, particularly polyacetylene in the mid-to-late 1970s, brought a new age of research in semiconducting polymer science [1]. These polymers have changed the scenario of nowadays electronics and display technology. During the last three decades, the tremendous progress in the field of semiconducting polymer materials has stimulated intensive research to achieve low-cost fabrication of devices with reasonable stability and performance. Despite a lot of published articles in this field, the potential for conduction in polymeric materials had been unknown for a long time. In usual saturated carbon-based polymers, the electrons are in a bound state and, therefore, are unavailable for electrical conduction. However, in conjugated polymers, conduction can be done by means of delocalized π-electrons. Conjugated polymers are known by their alternating single and double bonds. There is one π-orbital perpendicular to the plane of the macromolecule in addition to the in-plane σ-orbitals. The π-electrons are delocalized over several carbon atoms in the chain to form π-bands. The semiconducting nature of the polymer stems from the presence of an energy gap between the filled and empty bands. The delocalized π-electrons are mainly responsible for the electrical and optical properties in these systems [2]. Their semiconducting nature, easy structure modification, solution processability, flexibility, and cheap device solution fabrication such as spin-coating, dip-coating, and drop-casting make conjugated organic polymers a great candidate to be utilized in a broad range of organic electronic devices. Such applications can be organic thin-film transistors (OTFTs), polymer light-emitting diodes (PLEDs), chemical sensors and polymer solar cells (PSCs) [3,4,5,6,7,8,9].

Among the conjugated polymers, polyfluorenes and their copolymers have been extensively studied as a main class of emitting materials [10]. The ease of spin-coating or drop-casting solutions of polyfluorenes substituted with solubilizing side chains and the ability to tune a wide range of optical and electronic properties by copolymerizing basic fluorene monomers with other appropriately chosen conjugated units are considered as their significant features [11].

One of the best-known example of polyfluorenes is poly(9,9-dioctylfluorene-alt-benzothiadiazole) (F8BT), a yellow-green emissive copolymer. Its high luminescence quantum yield (60−80%), considerable ionization potential (∼5.9 eV), and relatively high electron affinity (∼3.3 eV) make this polymer a viable model choice to be utilized as an active layer and emitter material in OLEDs [12,13]. Although, in F8BT, the electron localization on the benzothiadiazole (BT) unit may decrease electron mobility, the mobilities of electrons and holes are similar as soon as the existing traps are filled. For thicker F8BT films aligned in the direction of current flow, holes and electron mobilities of 2.2 × 10^−3^ and 6.3 × 10^−4^ cm^2^/Vs were measured using a μFET (micro field effect transistor) [14]. A study on the correlation between molecular and the optoelectronic properties of F8BT cast films show that two main relaxation processes and the crystallization, which occurs at about 225 K (*β*-relaxation), 370 K (*α*-relaxation) and 470 K (crystallization) profoundly affect the electronic states [15]. Since the molecular geometrical restrictions are against hopping between neighboring sites, the conduction is trap-controlled below the glass transition. On the other hand, above the glass transition temperature, the trapped charge carriers become accessible by the backbone molecular motion, resulting in a structural de-trapping mechanism that dominates the charge conduction during and above *T_g_* [15]. Therefore, a practical and convenient approach to enhance the performance of PLEDs based on F8BT possessing electron transport characteristics can be post-annealing over the glass transition at 120 °C in a nitrogen atmosphere. This improvement may be attributed to the limited electron transport in F8BT films and the blocking of electron injection at the interface of F8BT/cathode [16]. A blend with 95% of F8BT and 5% (by weight) of F8T2has a remarkable feature in terms of hole transport and hole/electron balance. Therefore, it can be another method for increasing the efficiency of LEDs because of higher hole mobility of the blend with respect to neat F8BT [17]. According to previous studies on F8BT blends with other fluorene copolymers, polymer packing structures, especially interchain species formation, such as aggregate and excimer states, affect the optoelectronic and charge transport properties in conjugated polymer thin films. Moreover, the degree of crystallinity and orientation of the polymer chains with respect to the electrodes in a device structure are the parameters that play a vital role in charge carrier mobilities [18]. This correlation between particular polymer packing structures and charge transfer is manifested in the optical properties of the thin films. Therefore, a proper comprehension of the intermolecular (excitonic) coupling and electron-vibrational coupling concepts leads us to understand the charge and energy transport in soft organic assemblies. The electronic interactions that occur either within a given chain or between chains stem from the aggregates of the conjugated polymer. J-aggregates (H-aggregates) occur when “head-to-tail” (side-by-side) orientations dominate [19]. In J-aggregates (H-aggregates), the couplings are negative (positive), resulting in a spectral redshift (blue shift). Polaronic Frenkel excitons are responsible excitations for the optical response. It should be noted that, in Frenkel excitons, a field of vibrational excitations surrounds a central vibronic excitation. These excitons can be estimated by the multiparticle representation of Hamiltonians which describe exciton-vibrational coupling in organic assemblies [20].

In the Franck–Condon distortion: in H(J)-aggregates, there is a decrease (increase) in the vibronic peak ratio *R_abs_* with exciton bandwidth *W*, while *R_em_* increases (decreases) with increasing disorder and rising temperature. Here, *R_abs_* ≡ I_(0−0)_/I_(0−1)_ is the ratio of the oscillator strengths in the (0−0) and (0−1) absorption mode, the same for *R_em_* ≡ I_(0−0)_/I_(0−1)_ in emission mode. However, recognizing these two aggregate types using the usual spectral shifts can be misleading, especially if analyzing the photophysical response in more complex morphologies. For instance, lutein diacetate aggregates, which were already assumed to be J-like based on spectral shifts, are actually H-like, with *R_abs_* significantly decreasing with aggregation [20]. In addition, an improvement in intrachain planarization causes a significant redshift in the case of poly(3-hexylthiophene) (P3HT), despite its H-aggregate nature [19].

Donley et al. have shown that the optoelectronic and charge transport properties of F8BT spin-coated films have been affected by the restructuring of the packing structure resulting from various molecular weights upon annealing [18]. The considerable torsion angle between the F8 and BT mer units in pristine films changes upon annealing to adequately high temperature, resulting in an alternating packing structure in which the BT are separated from one another units in neighboring chains, which causes more complex energy and electron transfer. Therefore, the PL spectrum shows more emission from a high-energy state, and PL efficiencies enhance simultaneously [18]. Similarly, the packing structure of F8BT thin films that affects the triplet generation and dynamics causes a considerable increase in the triplet absorption through a solid-state restructuring of F8BT molecules. Therefore, it affects the interchain exciton migration efficiency, the efficiency of intersystem crossing and the monomolecular triplet lifetime [11].

Film thickness is a crucial parameter that impacts on the absorption, exciton formation, photoluminescence, exciton diffusion length, carrier mobility, and the thin conjugated polymer film’s microstructure because of its various nonlocalized (aggregate states) and localized (intrachain) transitions manifestations. With the increase of the polymer film’s thickness, the PL emission suddenly changes its character from prevailing intrachain interaction to the level of interchain interaction, and a mesoscale (between nano-and micro-dimensions) effect emerges due to more ordered microstructure. The competition between the formations of J- and H-aggregates could be the reason for this phenomenon. Based on the aforementioned facts, it appears that there is a thickness threshold in all of the investigated aspects of the PTB7, Poly [2-methoxy-5-(2-ethylhexyloxy)-1,4-phenylenevinylene] (MEH-PPV), and polysilanes thin films [21,22,23].

However, up to this date, only a few reports on the packing structure of F8BT thin films are available. In this article, we present a comprehensive study of structural ordering impact on the electric and optoelectronic properties of F8BT based on results achieved using the novel ER-EIS and other spectroscopic methods. All examined properties indicate a steep change in trends at approximately 100 nm film thickness. Therefore, we expect a “mesoscale effect” to be manifested with the threshold lying at the boundary between nano- and microscale. For the first time, this phenomenon is discussed in the case of F8BT in terms of structure packing and H- and J- aggregates formation.

## 2. Materials and Methods

**Figure 1:**
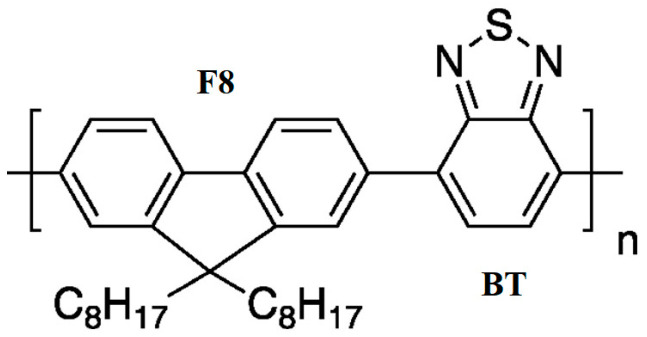
Schematic structure of F8BT polymer.

Poly(9,9-dioctyfluorene-alt-benzothiadiazole), F8BT material and toluene (p.a.) were purchased from Sigma-Aldrich (Prague, Czech Republic) and PENTA, Prague, Czech Republic, respectively, and used without further purification. F8BT chemical structure is depicted in Figure 1.

To provide films with thicknesses varying from tens to hundreds of nanometers, the solutions of polymer F8BT in toluene (100 μL) with concentrations ranging from 0.5–3.0% were deposited onto the quartz glass and ITO coated quartz glass substrates by spin coating at (1000, 2000, 3000, 4000, 5000) rpm in 30 s using Laurell WS-650-MZ-23 NPP (Laurell Technologies Corporation, North Wales, PA, USA). In order to prevent the effect of oxygen and moisture on the samples, all solutions and thin films were prepared in a glovebox. To obtain films with a thickness higher than 200 nm, F8BT solutions with 1.5% and 3.0% concentrations were cast onto the prepared quartz and indium tin oxide (ITO)-coated quartz substrates. All substrates were cleaned by successive 12 min sonication in distilled water plus Hellmanex, acetone and isopropyl alcohol, followed by UV ozone treatment for 10 min. Thicknesses of films on quartz for UV-VIS were 10, 45, 75, 95, 120, 165 nm, thicknesses of films on quartz/ITO substrates for photoluminescence measurement were 25, 45, 75, 105, 120, 230, 310, 360 nm and thicknesses of films on quartz/ITO for ER-EIS and GIWAXS were 27, 35, 60, 93, 135, 205, 310 nm, respectively. The UV-Vis absorption spectra were recorded on a Perkin–Elmer Lambda 1050 Spectrometer (Prague, Czech Republic). Fluorimeter FSL 920 equipped with high-voltage tungsten lamp from Edinburgh Instruments (Livingston, UK) was used for the measuring of PL spectra. Emission and excitation spectra were corrected to the light source and to the spectral sensitivity of the detector. A standard L geometry was used with a single-tilted specimen with a 55° incident angle of excitation light. All measurements were performed in vacuum in the cryostat (LN2 Optistat, Oxford Instruments, Bognor Regis, UK). The thickness was measured by mechanical profilometer with a 1 nm resolution (Dimension ICON, Bruker, Billerica, MA, USA).

The novel energy resolved-electrochemical impedance spectroscopy (ER-EIS) method [24,25] was used to map the density of states (DOS) in F8BT thin films. Although the experimental assessment of DOS is not all straightforward, the ER-EIS method was proved to be powerful not only for DOS mapping of organic semiconductors over five orders of magnitude and wide energy window [26], but also for investigation of effects of structural disorder, crystallinity in polymers [25,27], and charge generation, transfer, recombination and extraction in organic solar cells [27,28]. The ER-EIS method is based on the measurement of the charge transfer resistance, *R_ct_*, of a semiconductor/electrolyte interface at a frequency where the redox reactions determine the real component of the impedance [24]. The interaction between a thin organic film and an electrolyte via redox reactions in an electrochemical cell is similar to extraction of an electron by an acceptor and capture of an electron from a donor at a semiconductor surface through common solid state reactions [24]. Further, the DOS function *g*(*E*) in the semiconductor at the electrochemical potential *E_F,redox_* = *eU* is given in terms of *R_ct_* measured at the applied voltage *U* as [24]

(1) $$g\left(E_{F,redox}-eU\right)=\frac{1}{e^{2}k_{et}\left[A\right]SR_{ct}}$$

where *e* is the elementary charge, *k_et_* is the charge-transfer rate constant, [*A*] is the concentration of the electrolyte redox (donor/acceptor) species in the interphase region of the solid/liquid contact, and *S* is the sample area. The reciprocal value of the *R_ct_* resistance measured as a response to the harmonic perturbation with the amplitude d*U*, and an appropriate frequency provides direct information about the electronic DOS at the energy-adjusted using an external voltage. The impedance/gain-phase analyzer Solartron analytical, model 1260 (Ametek Scientific Instruments, Oak Ridge, TN, USA) was used for the ER-EIS experiment. The frequency was set to 0.5 Hz, the RMS value of AC voltage, d*U*, was 100 mV, and the average sweep rate of the DC voltage ramp was 10 mVs^−1^. The measurements were performed in a glove box with protective N_2_ atmosphere (oxygen and moisture below 20 ppm and 2 ppm, respectively) using a common three-electrode electrochemical cell with a volume of about 200 µL. The solution of 0.1M tetrabutylammonium hexafluorophosphate (TBAPF6) in acetonitrile was used as the supporting electrolyte. The potential of the working electrode with respect to the reference Ag/AgCl electrode was controlled via the potentiostat. Pt wire was used as the counter electrode. The potential recorded with respect to the reference Ag/AgCl electrode can be recalculated to the local vacuum level assuming the Ag/AgCl energy vs. vacuum value of 4.66 eV [24].

The GIWAXS (grazing-incidence wide-angle X-ray scattering) patterns were measured using the Nanostar SAXS/WAXS system (Bruker, Germany) equipped with a metal-jet X-ray source (Excillum, Sweden). The X-ray source generates GaKα radiation (*E* = 9.2 keV, λ = 1.34 Å). The photon flux is approximately 10^9^ photons/s. The final X-ray beam was shaped by Montel optics (Incoatec, Germany) and the resulting parallel beam had a divergence of 500 μrad in both horizontal and vertical directions. The X-ray scattering background was reduced by vacuating the path from the X-ray source to the X-ray detector. The angle of incidence of X-ray beam on the polymer sample was set to 0.2°. The sample-detector distance was set to 219 mm. The GIWAXS patterns were recorded for 600 s by 2D X-ray detector Pilatus 300K (Dectris, Switzerland). The pixel size of the X-ray detector was 172 × 172 μm2 (H × V).

## 3. Results and Discussion

### 3.1. UV-Vis Spectroscopy

**Figure 2:**
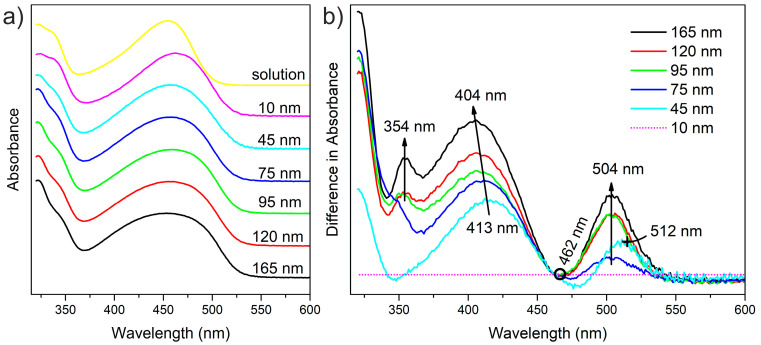
Graph (**a**) UV-Vis spectra of F8BT with different thicknesses on glass substrates. Graph (**b**) Difference between the spectra for thin films and the spectrum of the thinnest one. Magenta dot line indicates zero difference for the 10 nm sample itself.

The UV-Vis spectra of F8BT films with different thicknesses and in the solution in toluene are depicted in Figure 2a. It can be noted from the graph that the main absorption peak is observed at about 460 nm. The spectra are normalized to the absorbance value at 462 nm. This main absorption band in the spectra is caused by π-π* electron transitions of π-delocalized electrons along the polymer chain, mainly along the F8 unit [29]. With increasing thickness, the absorption spectra show a tiny blue shift of maxima less than 10 nm, where the absorbance maximum, for film with thickness 10 nm, is 462 nm; in the case of thicker films, the maximum is at 454 nm. The maximum for F8BT solution is observed at 455 nm. Moreover, the absorption band broadens slightly with increasing thickness of films. The changes and order development can be better observed in Figure 2b, showing difference between the spectra of films and the thinnest (10 nm) film. The reference point marked by an empty circle corresponds to the absorbance at the maximum wavelength for 10 nm film and was used for spectra normalization. On the other side, the peak centered at 504 nm was observed, which indicates broadening of the absorption peak in the original spectra. The 45 nm thick film shows maximum broadening at 512 nm as the only exception. A blue shift of the maximum is best manifested for films 45 nm and 75 nm thick by the negative valleys right to the reference point. The blue shift for other films is outweighed by the broadenings. The maximum broadening at the blue side of the absorption peak increases in intensity from 413 nm to 404 nm. Moreover, a fine feature is formed at 354 nm, which cannot be observed in thinner films. These changes may be caused by the formation of a new energy state due to interchain aggregate states between the polymer chains [16]. On the other hand, if one can expect that the formation of H-aggregates is more favored in thicker films, the absorption spectra would preferentially broaden toward longer wavelengths. This contradiction can be explained by the creation of JH-aggregates, where the interplay of these two aggregation modes results in the broadening towards higher energies. A similar explanation has been proposed by Donley in [16], where it is described as the alternating π-stacking of polymer chains.

### 3.2. Photoluminescence (PL) Spectroscopy

**Figure 3:**
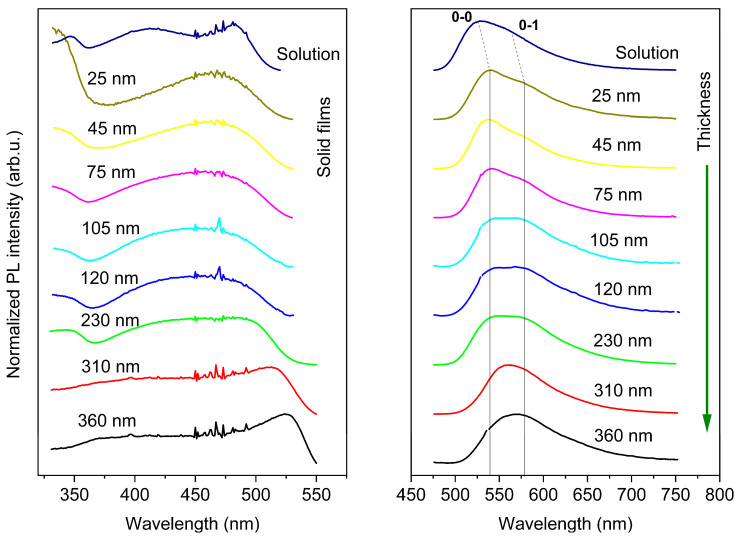
Excitation (**left**), λ_em_ = 576 nm and emission (**right**), λ_ex_ = 466 nm spectra of F8BT solution and films with different thicknesses.

The representative room temperature photoluminescence (PL) spectra of F8BT films are shown in Figure 3. Excitation spectra (left side) indicate that the positions of excitation maxima and the shape of the spectra are dependent on the thickness of the films. A slight blue shift and considerable broadening of these peaks are evident with increasing thickness varying from 45 nm to 120 nm. In the case of the thickest film, a significant change of spectrum shape and redshift of excitation maxima were observed. These phenomena can be plausibly explained by the extension of the conjugation length along the polymer chain. The longer the length of conjugated segments, the higher delocalization of exciton and the more excitation maxima shifted towards lower energy of transition vibrational levels in excited states [23].

From the emission spectra in the right graph, it can be seen that 0–0 (peak at higher energy for exciton recombination caused by the transition from the 0th vibrionic excited state to the 0th vibrionic ground state) peak is slightly red-shifted with increasing thickness. The spectral intensity for the second peak (marked as 0–1, peak at lower energy exciton recombination from the 0th vibrionic excited state to the 1st vibrionic ground state) relatively increases with increasing thickness. The 0–0 PL transition of the thinnest film is situated at 537 nm. For the films ranging from 45 nm up to 200 nm, the emission maxima of 0–0 transition are shifted towards 545 nm consecutively. For the films with a thickness higher than 200 nm, the 0–0 transitions almost disappear from the spectrum, and the 0–1 transition is prevailing. The shift of 0–0 transition can be attributed to a change of polymer chain packing [16], where the J-aggregation of chains supports this transition. Nevertheless, with the increasing thickness, the polymer chains can form a film with a more organized structure and, thus, the H-aggregation with alternating π-stacking of polymer chains is more favorable and changes the spectral features of the material. For the thickest films, the structural ordering of chains causes prevailing 0–1 with longer conjugation length resulting in low energy radiative states [16,21].

**Figure 4:**
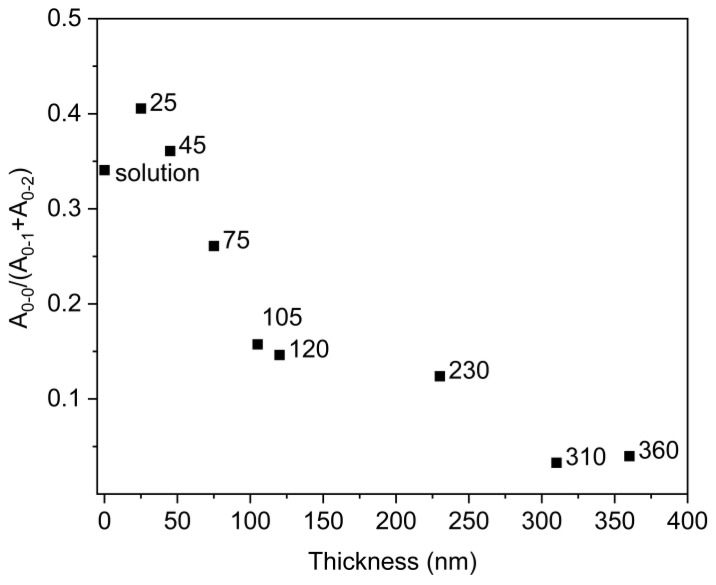
The dependence of peak area ratios of A_0–0_/(A_0–1_+A_0–2_) transitions on the thickness.

The PL emission spectra were analyzed in more detail. The emission peaks (0–0, 0–1 and 0–2) were deconvoluted and the area under peaks was integrated. The deconvoluted spectra are shown in Figure S1 in Supplementary Materials. A ratio of peak areas of 0–0, 0–1 and 0–2 PL transitions [A_0–0_/(A_0–1_+A_0–2_)] is introduced; the dependence of this ratio is shown in Figure 4. It is clearly seen that the ratio is steeply decreasing with the increasing thickness up to about 120 nm. For the samples with higher thickness, the ratio is close to the value 0.1; for the thickest sample (310 and 360 nm), the ratio almost approaches the value 0.

This behavior can reveal that, in the structure of thinner films (below 120 nm), the prevailing organization of polymer chains is in J-aggregation because this structural ordering is related to the radiative transition from the lowest exciton state to the vibrational ground state. On the other hand, in case of thicker films (over 120 nm), the 0–1 transition starts to dominate. This can indicate another structural ordering more favorable to H-aggregation and lowering of potential energy [30,31].

### 3.3. Density of State

The electronic structure in terms of density of state function (DOS) of F8BT films was measured by ER-EIS method.

The energy separation between highest occupied molecular orbital (HOMO) and lowest unoccupied molecular orbital (LUMO) in an organic solid is called HOMO–LUMO gap. The charge injection processes require promotion of an electron or a hole from the electrodes into one of the charge transport (HOMO or LUMO) states of the organic film. The transport gap, *E*_t_, which is the energy necessary to create a separated electron–hole pair, thus, offering a substantial polarization energy contribution (1–2 eV). Still, it exceeds the optical gap, *E*_opt_, by ∼1 eV. The optical gap corresponds to the formation of a Frenkel exciton with the electron and hole on the same molecule or a charge transfer exciton with the electron and hole on two adjacent molecules. The calculated difference between *E*_t_ and *E*_opt_ should be close to the exciton binding energy which can be as large as 1 eV. This difference between optical and transport gap is usually ignored for inorganic semiconductors due to the low Wannier exciton binding energy. For the inorganic semiconductor, the band gap can, thus, often be obtained from the onset of the optical absorption spectra and *E*_t_ equals, in a good approximation, the optical gap, *E*_opt_ [32].

The DOS was calculated from the measured resistance, *R*_ct_, using Equation (1), considering the concentration at the HOMO and LUMO in the order of 10^21^ cm^−3^eV^−1^.

**Figure 5:**
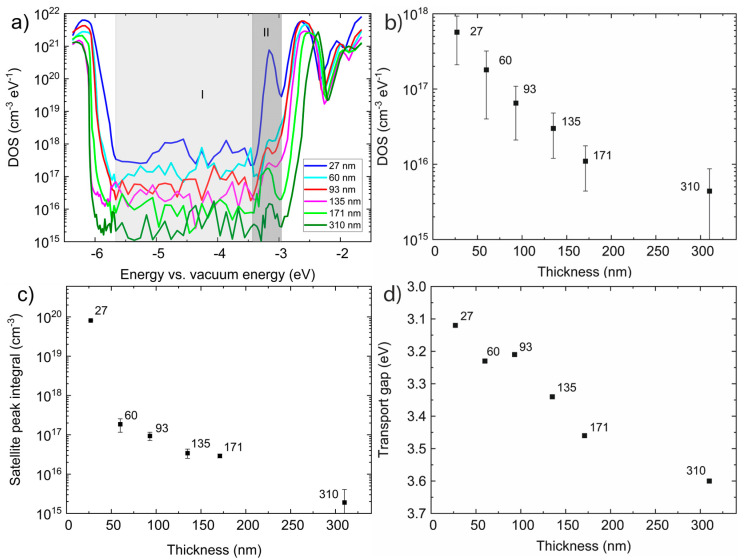
(**a**) ER-EIS spectra of F8BT films, (**b**) the dependences of DOS function on the F8BT film thickness, (**c**) the dependence of integral of satellite peak on the film thickness, (**d**) the dependence of transport gap on the film thickness.

The DOS spectra (Figure 5a) imply that hopping charge transport via the Gaussian distributed localized states is the predominant transport pathway [18]. It is clearly seen that the DOS are decreasing in the band gap region (light grey area I) with increasing film thickness. This decrease of DOS as a function of film thickness is depicted in Figure 5b. It can be explained by a general increase in structural ordering of polymer chains and the formation of organized units resulting in the lower population of defect states in the gap [21].

Other significant features observable in grey area II (Figure 5a are shallow states present 0.5 eV below the LUMO (−2.65 eV) in the thinner polymer films. The integrated area of these satellite peaks is plotted against the film thickness in Figure 5. The presence of the shallow states is, with high probability, due to the introduction of polymer chain packing disorder which may deteriorate the alternating structure of adjacent BT and F8 mer units in neighboring polymer chains [18]. Such structural feature should enhance the interchain electron transfer (hopping) through the population of states at ca −3.15 eV.

**Figure 6:**
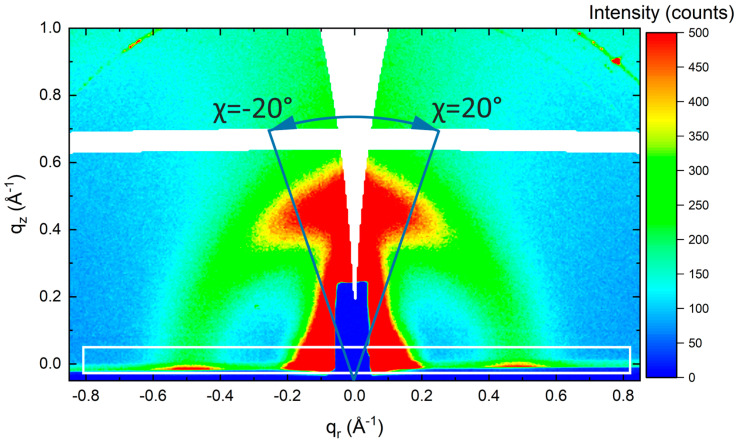
The representative GIWAXS pattern of a polymer layer with a thickness of 310 nm showing the integration area of the horizontal diffraction peaks along *q_r_* axis (white rectangle) and the principle of integration of the vertical diffraction peak along *q_z_* axis between *χ* = −20° and *χ* = 20°. The GIWAXS patterns of other thin films are in supplementary file.

**Figure 7:**
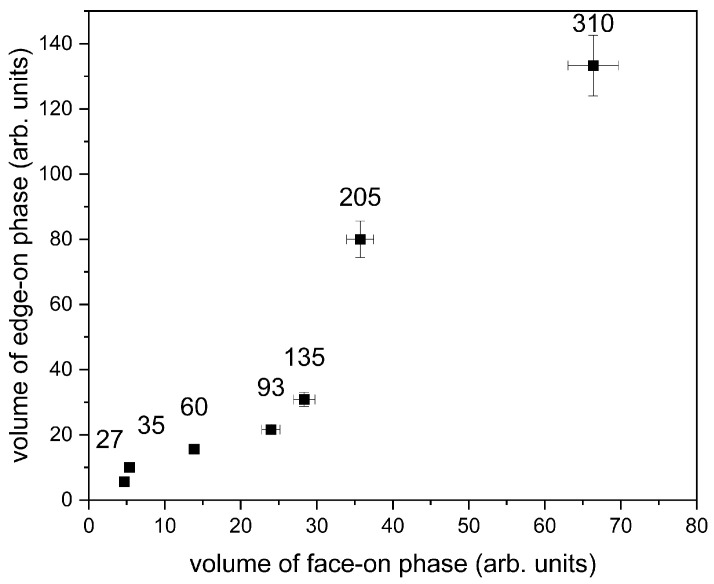
Dependence of volume phases on the thickness. The numbers above the points indicate the film thickness.

In Figure 5d, the dependence of the transport gap on the thickness is depicted. As mentioned above, the charge transport path is rather of a hopping nature and intrachain electron transport in F8BT is not expected to be a very efficient process, due to the strong localization of electrons in the LUMO on the BT units and the high energy barrier that the F8 sites would present as an electron moves along the chain [18]. For that reason, the ideal molecular orientation would be edge-on (side chain). From the GIWAXS measurement (Figure 6) and the analysis of measured data (Figure 7), it is obvious that the structural edge-on phase in films increases with thickness, implying a more organized structure in thicker films. One could expect that the transport gap will be narrower in case of thicker films. However, unexpectedly, the transport gap increases with increasing film thickness and ordering chains. This could be explained in terms of a degree of energetic disorder originating in a remarkable resilience of the backbone conformation to side-chain disorder, which causes the interaction of charge carrier with the environment over longer distances on the main chain and breaks the coherent transport and contributes to activation of hopping transport prevailing in F8BT [18,33,34]. Moreover, structural disorder is linked to electronic localization within molecular stacks and gives rise to electronic traps, which limit charge transport in high-mobility conjugated polymers. In high-mobility polymers, the fraction of the film comprised of ordered material is sufficiently large to be interconnected by bridging polymer chains, creating a network that sustains efficient charge transport. Hence, in high-molecular-weight polymers, transport occurs through an interconnected network of ordered regions. The amorphous fraction of the film does not participate in transport; therefore, the structural properties of the ordered regions govern charge transport in the film. Structural disorder gives rise to energetic disorder, that is, variations in the energy levels across the material, and also affects intermolecular charge transfer integrals. Disorder in the π-stacks of organic semiconductors is most relevant to transport because this direction supports rate-limiting intermolecular charge transfers [35]. Manifestation of a similar behavior and generation of side-chain disorder can also be induced by the addition of nanoparticle filler into the thin film. In such a case, better transport properties and better charge carrier recombination in LED devices were observed and explained in [36,37,38].

### 3.4. Structural Characterization—GIWAXS

The horizontal peaks along the *q_r_* axis in GIWAXS pattern in Figure 6 were integrated between *q_z_*=−0.01 Å^−1^ and *q_z_*=0.05 Å^−1^. Here, the horizontal peaks represent face-on orientation. The vertical peaks along the *q_z_* axis were integrated between *χ* values of −20° and 20°. The principle of vertical peak integration is schematically depicted in Figure 6. The polar angle *χ* represents the angle between the *q* scattering vector and the *q_z_* axis. Here, the vertical peaks represent edge-on orientation. As a result, Figure 7 shows the volume of the edge-on oriented phase plotted against the volume of the face-on oriented phase. Although not calibrated, it can be seen that the volume of the edge-on phase increases with the film thickness in a constant ratio with the face-on phase and the slope of this dependence changes at about 100 nm in favor of higher proportion of the edge-on phase. This observation suggests that the formation of the edge-on phase is more favorable in thicker films.

## 4. Conclusions

In summary, there is a clear correlation between the ratio of emission peaks dependence and the presence of a satellite peak in DOS spectra and the film thickness. The PL 0–1 transition rises with increasing thickness, what could be interpreted by diminution of the satellite DOS peak below LUMO, which causes a non-radiative transition instead of 0–0 transition. The increasing transport gap in the case of thicker films can be caused by lower structural disorder leading to coherent charge transport along the main chain, which is weaker than hopping in F8BT films. Crystalline phases with face-on and edge-on orientation of F8BT chains were identified in the films. The change in electronic structure correlates with the transition in the edge-on to face-on phase volume ratio, indicating an increasing trend for the edge-on orientation in thicker films. The principal difference between thinner and thicker films than ca 100 nm can be found in mutual interplay between J- and H-aggregation modes in the film structure. While the effect of J-aggregation prevails in thinner films, thicker films are dominated by H-aggregation, with alternating π-stacking of F8 and BT units, which is favorable for increased structural ordering.

We observed a thickness threshold in all investigated aspects of the films at the thickness lying between nano- and microscale at about 100 nm. Such pronounced presence of the threshold suggests that utilization of solution spin-coating and possibly other solution deposition techniques gives different results depending on whether thinner or thicker films shall be prepared. Although a specific threshold value may be expected for each combination of polymer, solvent and process conditions, the value of about 100 nm seems to be the typical length scale of this phenomenon in general.

Our results and findings could lead to a broadening of knowledge in the field of electronic devices fabrication, as it would be possible to predict the behavior and critical parameters such as charge transport, exciton diffusion length, luminescence for a broad range of applications, and to the observation of physical phenomena that have hitherto been prevented by disorder-induced localization.

## Data Availability

The data presented in this study are available on request from the corresponding author.

## Supplementary Materials

The following supporting information can be downloaded at: https://www.mdpi.com/article/10.3390/polym14030641/s1, Figure S1. Deconvoluted PL emission peaks with their integrated areas.
