# Supplementary material for: Thickness Dependence of Electronic Structure and Optical Properties of F8BT Thin Films
Source: Polymers (Basel). 2022 Feb 8;14(3):641. doi: 10.3390/polym14030641 (PMC8838540; doi:10.3390/polym14030641)
Supplement: Supplementary file 1 [file polymers-14-00641-s001.zip › polymers-1571243-supplementary.pdf]

polymers

Supplementary Information

Thickness dependence of electronic structure and optical properties of F8BT thin films

Bita Ghasemi [1], Jakub Ševčík [1], Vojtěch Nádaždy [2], Karol Végsö [2], Peter Šiffalovič [2], Pavel Urbánek [1,]* and Ivo Kuřitka [1]

[1] Tomas Bata University in Zlin, Centre of Polymer Systems, Tr. Tomase Bati 5678, Zlin, CZ-760 01, Czech Republic
[2] Institute of Physics, Slovak Academy of Sciences, Dubravska cesta 9, Bratislava, SK-845 11, Slovak Republic
* Correspondence: Correspondence: urbanek@utb.cz

Deconvolution of emission peaks

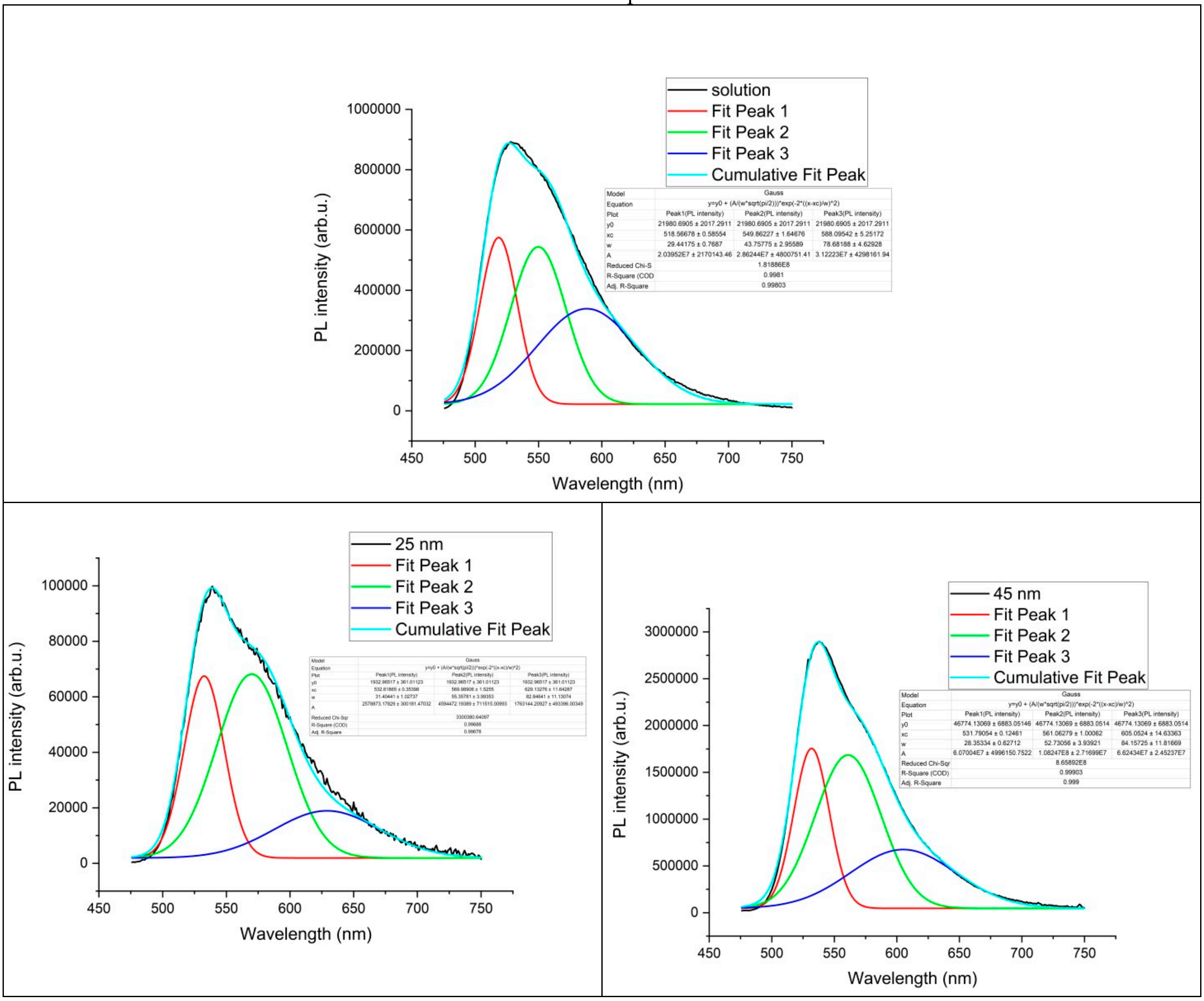

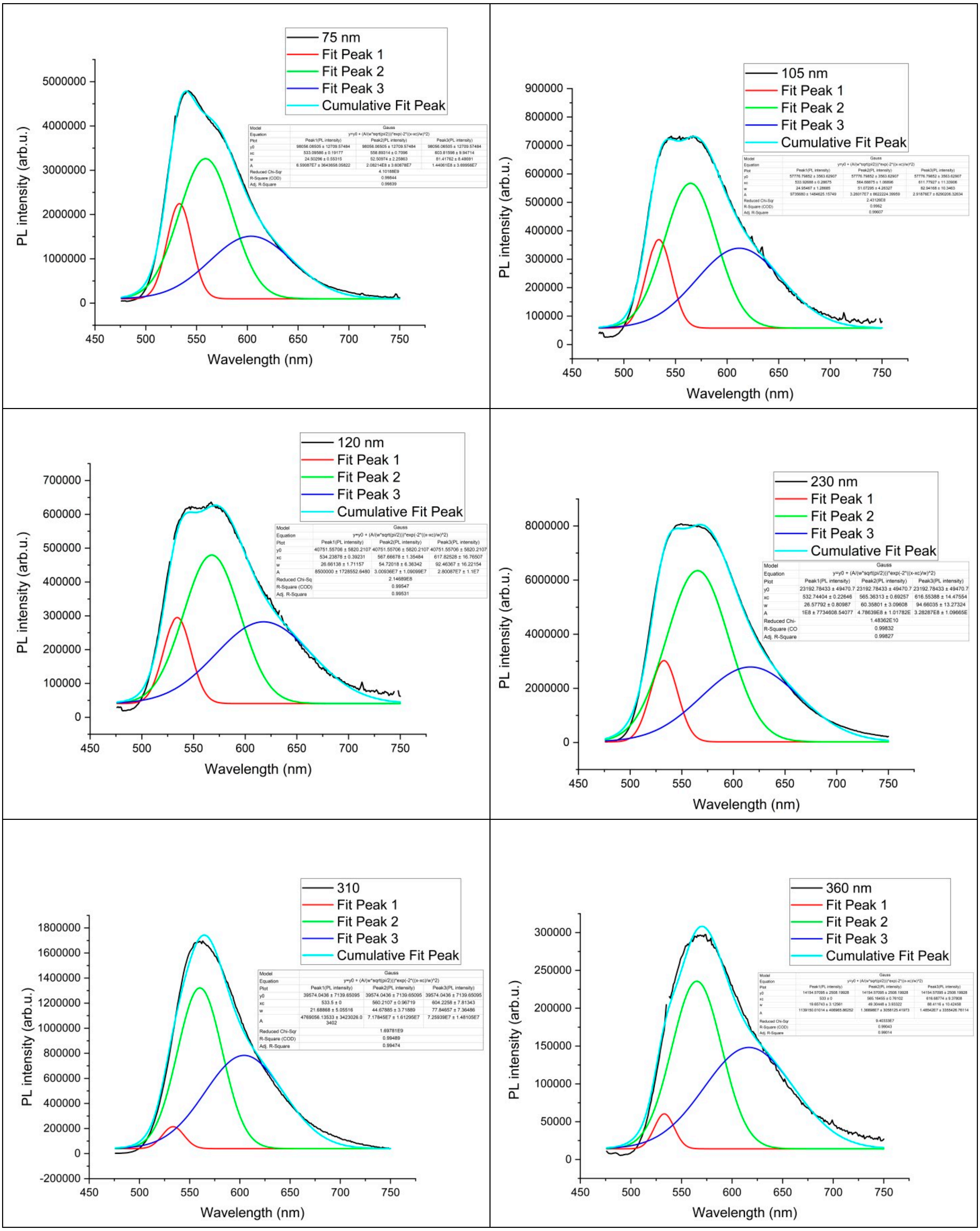

Figure S1 Deconvoluted PL emission peaks with their integrated areas.

GIWAXS Patterns

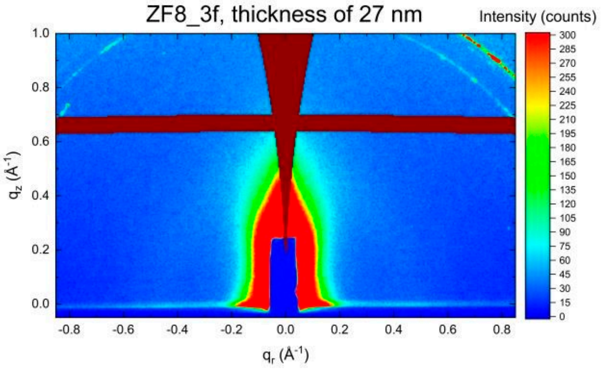

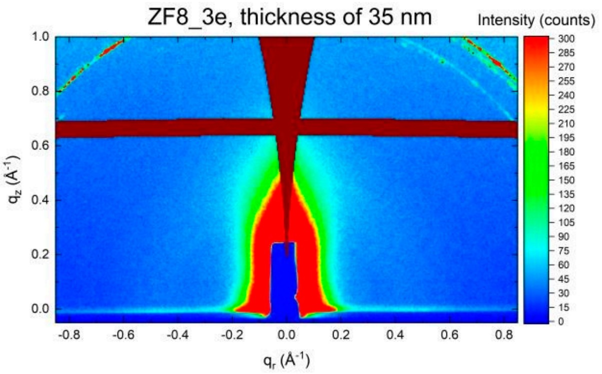

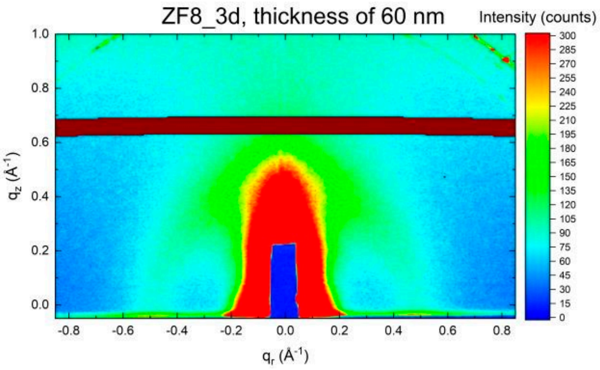

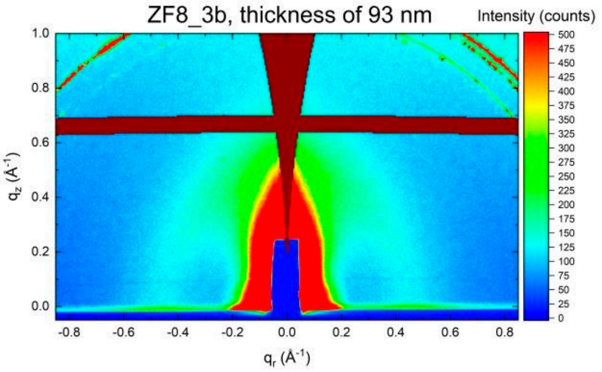

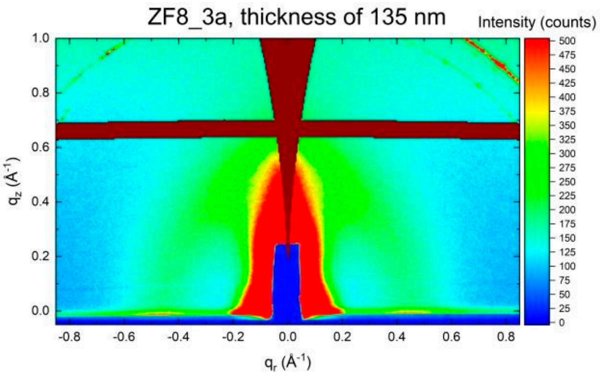

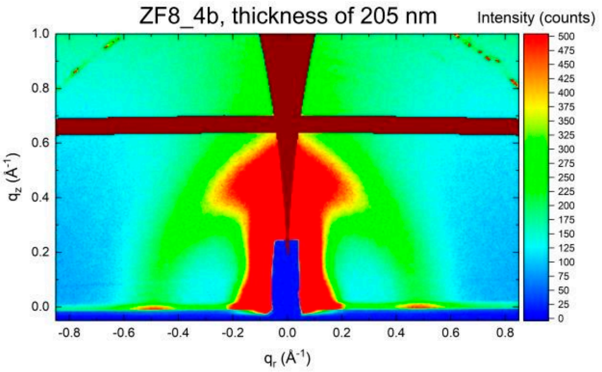

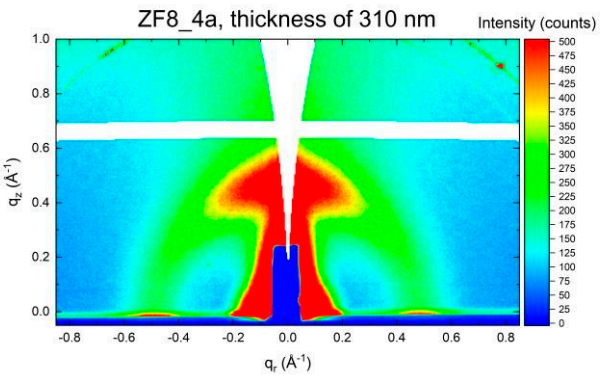

Figure S2 The GIWAXS pattern of polymer layers showing the integration area of the horizontal diffraction peaks along q_r axis (white rectangle) and the principle of integration of the vertical diffraction peak along q_z axis between χ= -20° and χ= 20°.
